# Safety, efficacy, and survival of drug-eluting beads-transarterial chemoembolization vs. conventional-transarterial chemoembolization in advanced HCC patients with main portal vein tumor thrombus

**DOI:** 10.1186/s40644-023-00581-8

**Published:** 2023-07-22

**Authors:** Junwei Chen, Lisha Lai, Churen Zhou, Junyang Luo, Haofan Wang, Mingan Li, Mingsheng Huang

**Affiliations:** 1 Department of Interventional Radiology, the Third Affiliated Hospital of Sun Yat-Sen University, Guangzhou, 510630 China; 2 Department of Radiology, School of Medicine, Guangzhou First People’s Hospital, South China University of Technology, Guangzhou, 510180 China

**Keywords:** Hepatocellular carcinoma, Main Portal vein tumour thrombus, Transarterial chemoembolization, Microspheres, Survival analysis

## Abstract

**Objectives:**

To compare the efficacy, overall survival (OS) and safety of drug-eluting beads-TACE (DEB-TACE) and C-TACE as initial treatment in advanced hepatocellular carcinoma (HCC) patients with main portal vein tumor thrombus (mPVTT).

**Methods:**

The medical records of consecutive advanced HCC patients with mPVTT who underwent initial DEB-TACE or C-TACE from September 2015 to October 2021 were retrospectively evaluated. Treatment crossover was allowed in this retrospective research. The adverse events, disease control rate (DCR), time to tumor progression (TTP) and OS of patients who underwent DEB-TACE were compared with those of patients who underwent C-TACE.

**Results:**

Eighty-three patients were included: 42 patients in DEB-TACE group and 41 patients in C-TACE group. DEB-TACE could be safely performed in HCC patients with mPVTT, and they gained a better DCR than those submitted to the C-TACE (76.2% vs. 53.7%, *P* = 0.031), which might have resulted in longer TTP (median TTP: 9.0 months vs. 3.0 months, *P* < 0.001). Furthermore, DEB-TACE showed significant OS benefits compared with C-TACE (median OS: 12.0 months vs. 5.0 months, *P* < 0.001). DEB-TACE, absence of arterioportal shunts (APS), leisons with capsular non-infiltration were found to be independent prognostic factors for better OS. Furthermore, subgroup analysis proved that patients with good DCR gained longer OS in DEB-TACE group.

**Conclusions:**

DEB-TACE could be safely performed and improve the DCR of HCC patients with mPVTT, which resulting in longer TTP and OS, compared with C-TACE.

**Supplementary Information:**

The online version contains supplementary material available at 10.1186/s40644-023-00581-8.

Hepatocellular carcinoma (HCC) is the sixth most common malignant tumor and the third most common cause of tumor-related deaths worldwide [1, 2]. Approximately 10–40% of HCC patients are diagnosed at an advanced stage with main portal vein tumor thrombus (mPVTT). The mPVTT is possibly associated with poor prognosis, and it is supposed to increase the risk of wide tumor transmission and the pressure of the portal vein (causing variceal hemorrhage, ascites and liver failure) [3]. The median overall survival (OS) of these patients is only 2.7–4.0 months if left untreated [4].

Systemic therapy (tyrosine kinase inhibitor-TKI and immune checkpoint inhibitors-ICIs) is currently regarded as the standard of care for advanced-stage HCC patients [5]. However, data supporting the survival benefits from systemic therapy among patients with mPVTT are still limited. It is well known that conventional-transarterial chemoembolization (C-TACE) can be used as a palliative treatment for intermediate/advanced stage HCC according to several guidelines [6, 7]. Zhang et al. had reported that C-TACE could improve the OS in patients with the PVTT compared to sorafeinib [8]. Furthermore, C-TACE is supposed to prolong OS in advanced HCC with mPVTT [9]. However, C-TACE remains a challenge for patients with mPVTT because of the risk of deteriorating hepatic function due to ischemic liver damage [4].

Drug-eluting beads-TACE (DEB-TACE), an advanced technology, uses particulate agents of different sizes formed by permanent embolization hydrogels that slowly release chemotherapeutic drugs into HCC tissues and minimize the blood concentration of chemotherapeutic drugs and related systemic effects, consequently improving safety and efficacy compared with C-TACE [10, 11]. Due to the properties of DEB-TACE, it is possible to further improve the clinical benefits of HCC with mPVTT compared to C-TACE, which has not been demonstrated. Therefore, we performed this retrospective study to investigate safety and efficacy of DEB-TACE in HCC patients with mPVTT and to compare with C-TACE.

## Material and Methods

### Baseline status

This retrospective study was approved by the Institutional Review Board (IRB) in accordance with approved guidelines of our institution. Due to the retrospective nature of the study, the IRB waived the need for written informed consent. Patients were eligible if they had: (1) imaging or pathological diagnosis of HCC; (2) mPVTT confirmed by the detection of the enhancement of an intraluminal mass expanding the main portal vein on the arterial phase and a low-attenuation, intraluminal mass on the portal phase by contrast-enhanced computed tomography (CT) or magnetic resonance imaging (MRI); (3) preserved liver function (Child–Pugh classes A and B7); and (4) an Eastern Cooperative Oncology Group (ECOG) performance status of 0–1.

The population comprised a single-institution, retrospective cohort including 204 patients diagnosed with mPVTT treated with DEB-TACE or C-TACE from September 2015 to October 2021. Therefore, the choice of TACE has been decided case-by-case within the multi-disciplinary treatment board (consisting of interventional radiologists, medical oncologists and liver surgeons), and by a discussion with the patient himself/herself. Before the initial TACE, the interventional radiologists recommended patients choose either DEB-TACE or C-TACE, and they signed informed consent for DEB-TACE or C-TACE.

Patients were excluded if they had: (1) other malignant extrahepatic diseases; (2) previous surgery or other local–regional therapies (radiofrequency ablation, I125-seed implantation, etc.); (3) acceptance of hepatic artery infusion chemotherapy; (4) other serious medical comorbidities; (5) contraindications to carboplatin, epirubicin, lipiodol or TACE procedures.

### Protocol for DEB-TACE/C-TACE

Main TACE interventional radiologists (IRs) (JW.C, MA.L and MS.H) were highly experienced with more than 15 years of experience in TACE treatment, respectively. Tumor-feeders artery (both intra-hepatic and extra-hepatic) were carefully identified on the artertial phase by contrast-enhanced CT/MRI before TACE. Angiograph was performed using a 5-F RH catheter or Cobra catheter (Cook) and a 2.4-F to 2.8-F microcatheter (Renegade, Boston Scientific; Master PARKWAY HF, Asahi; Merit Maestro Microcatheter, Merit Medical) superselectively toward the tumor-feeding arteries. And mPVTT could be identified through the tumor staining and contrast reflux under DSA, which could be confirmed with contrast-enhanced CT of mPVTT location. The embolization of mPVTT could be performed in patients without arterio-portal shunts (APSs) at first step during the TACE procedure. In patients with APS, portal supply could be visualized in early phase under fluoroscopy, embolization using Embosphere microspheres (300–500 or 500–700 µm, Embosphere, Merit Medical), which were diluted two times with contrast medium, was performed superselectively to occlude the arteries to APSs before chemoembolization in both groups until stasis confirmed by post-embolization angiography.

**Fig. 1:**
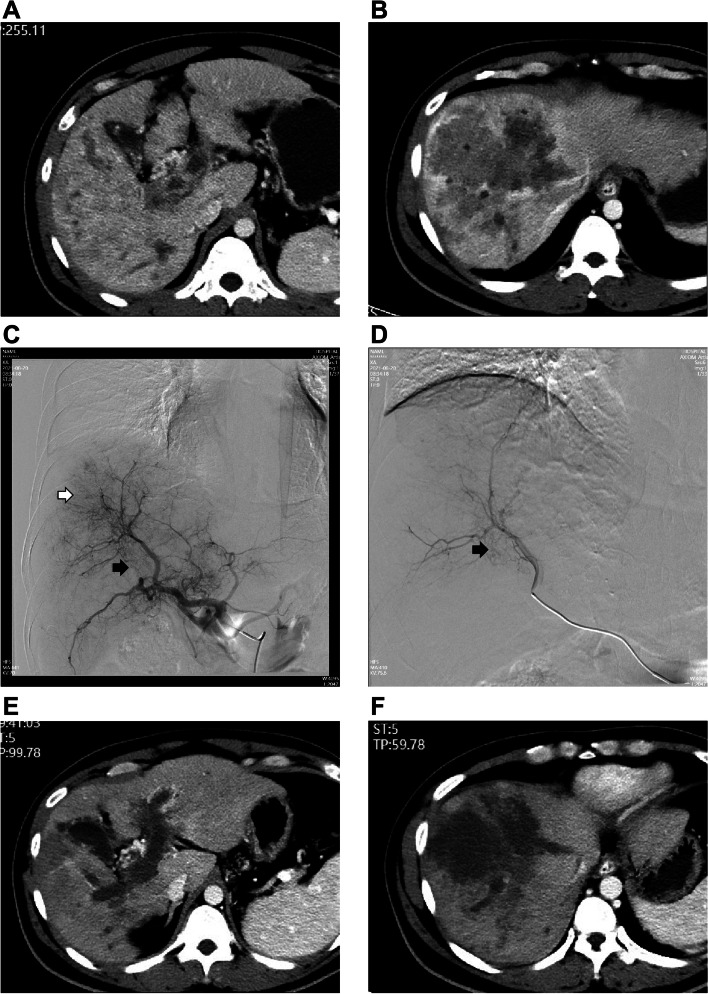
Superselective embolization TACE procedure and follow-up in A 40-year-old male HCC patients with mPVTT. **A**,** B** contrast-enhanced CT scan showed a HCC lesion (maximal diameter of 110 mm) with main portal vein invasion. **C** DSA of common hepatic artery during the first DEB-TACE procedure (Black Arrow: mPVTT, White Arrow: Tumor leision). **D** superselective embolizations of tumor feeding artery during DEB-TACE procedure (Black Arrow: mPVTT). **E**,** F** contrast-enhanced CT scan showed PR in 1 month follow-up. mPVTT = main portal vein tumor thrombus; DSA = Digital subtraction angiography; DEB-TACE = Drug Eluting Beads-TACE

Second step, for the DEB-TACE group, depending on the tumor burden, vessel size, shunt and PVTT involvement, appropriate diameter of HepaSphere microspheres (30–60 µm or 50–100 µm, Merit Medical) were loaded with 30–50 mg of doxorubicin hydrochloride. For example, small size (≤ 5 cm), without APSs, 30–60 µm HepaSphere were prepared. 50-100um HepaSphere would be adopted for large hypervascular tumor (> 5 cm) and with APSs. HepaSphere microsphere were then injected into the tumor-feeding artery superselectively and slowly under free flow (Fig. 1). The embolization endpoint was defined as stasis of blood flow in the tumor-feeding artery, and repeated hepatic arteriography was performed to assess the devascularization after each embolization step in the DEB-TACE procedure. Meanwhile, for the C-TACE group, an emulsion of 2–10 mL of lipiodol (Lipiodol Ultrafluide, Guerbet) with 20–60 mg of doxorubicin hydrochloride (Pfizer) (ratio of lipiodol/doxorubicin hydrochloride was 2:1) was also injected superselectively and emulsion was usually prepared using the pumping technique through a three-way stopcock [12]. The dosages of lipiodol and doxorubicin were determined by tumor size, vascularity, presence of APSs and underlying liver function, less than 10 ml of lipiodol for C-TACE was used in most C-TACE procedure in our research (Supplement Fig. 1). If the embolization endpoint was not reached, gelatin sponge particles (Cook), which were mixed with contrast material, were administered into the feeder vessels until stasis in both groups.

### Follow-up and repeated TACE procedure

All patients were required to undergo follow-up, in accordance with our institutional protocol. Each follow-up session included a detailed history and physical examination, laboratory tests, and contrast material-enhanced CT/MR. Follow-up was conducted at 4- to 8-week intervals after previous TACE. Patients with intrahepatic residual viable tumors or recurrent tumors on CT/MR underwent repeated TACE if the Child–Pugh status remained at class A or B and there was no evidence of hepatic decompensation (uncontrolled ascites or hepatic encephalopathy). IRs encouraged patients to stick to initial TACE procedure but patients could still choose either DEB-TACE or C-TACE, and they signed informed consent for DEB-TACE or C-TACE during the follow-up. Subsequent treatment crossover (eg, following C-TACE in the DEB-TACE group and following DEB-TACE in the C-TACE group) was allowed. Combined Sorafenib (400 mg Bid) or lenvatinib (8 mg Qd) were allowed when patients were diagnosised as PD in the follow up period in both groups.

### Assessment

The clinical, laboratory, and radiologic records were reviewed. The safety assessment included documentation of clinical laboratory tests, physical examinations, and measurements of vital signs. In the two groups, liver function tests within 7 days after the first TACE procedure were recorded and adverse events (AEs) within 1 month were reported according to the Society of Interventional Radiology guidelines and graded according to Common Terminology Criteria for Adverse Events (CTCAE) version 4.0 [13, 14].

The radiologic records obtained during follow-up were assessed in consensus by two radiologists with more than 10 years of experience in abdominal diagnosis. Tumor responses were assessed by contrast-enhanced CT/MRI at 4–8 weeks after the initial TACE procedure, using the modified Response Evaluation Criteria in Solid Tumors (mRECIST) [15]. The disease control rate (DCR) was defined as the percentage of patients who had the tumor response ratings of complete response (CR) or partial response (PR), or stable disease (SD). Time to tumor progression (TTP) was defined as the time from the first TACE procedure to progresson disease (PD) or any kind of death in the absence of confirmed progression, and patients did not diagnose PD at the end of follow-up were recorded as censored.

OS was defined as the time from the first TACE procedure to death or the last follow-up, and patients alive at the end of follow-up were recorded as censored in both groups. Patients were stratified on the basis of ChildPugh class, AFP value, tumor characteristics (including: APS, hepatic vein invasion (HVI), extrahepatic spread (EHS), maximum diameter, number and capsular) and DCR after initial TACE.

### Statistical analysis

All statistical analyses were performed using SPSS (IBM SPSS Statistics for Windows, version 19.0, IBM Corp.). Quantitative data are reported as the mean ± SD and were compared between these two groups using continuity correction and the independent-samples *t* test, Pearson’s x^2^ test, and Fisher’s exact test. Categorical data were compared using the x^2^ test. The Kaplan–Meier method and log-rank test were used to estimate and compare TTP and OS, respectively, and corresponding 95% confidence intervals (CIs) were reported. Variables with a *P-*value less than 0.10 in univariate analysis were entered into a multivariate analysis. Multivariate analyses were performed with a Cox proportional hazard regression model. All *P-*value calculations were two sided, and *P* < 0.05 was considered statistically significant.

## Results

### Baseline information

**Fig. 2:**
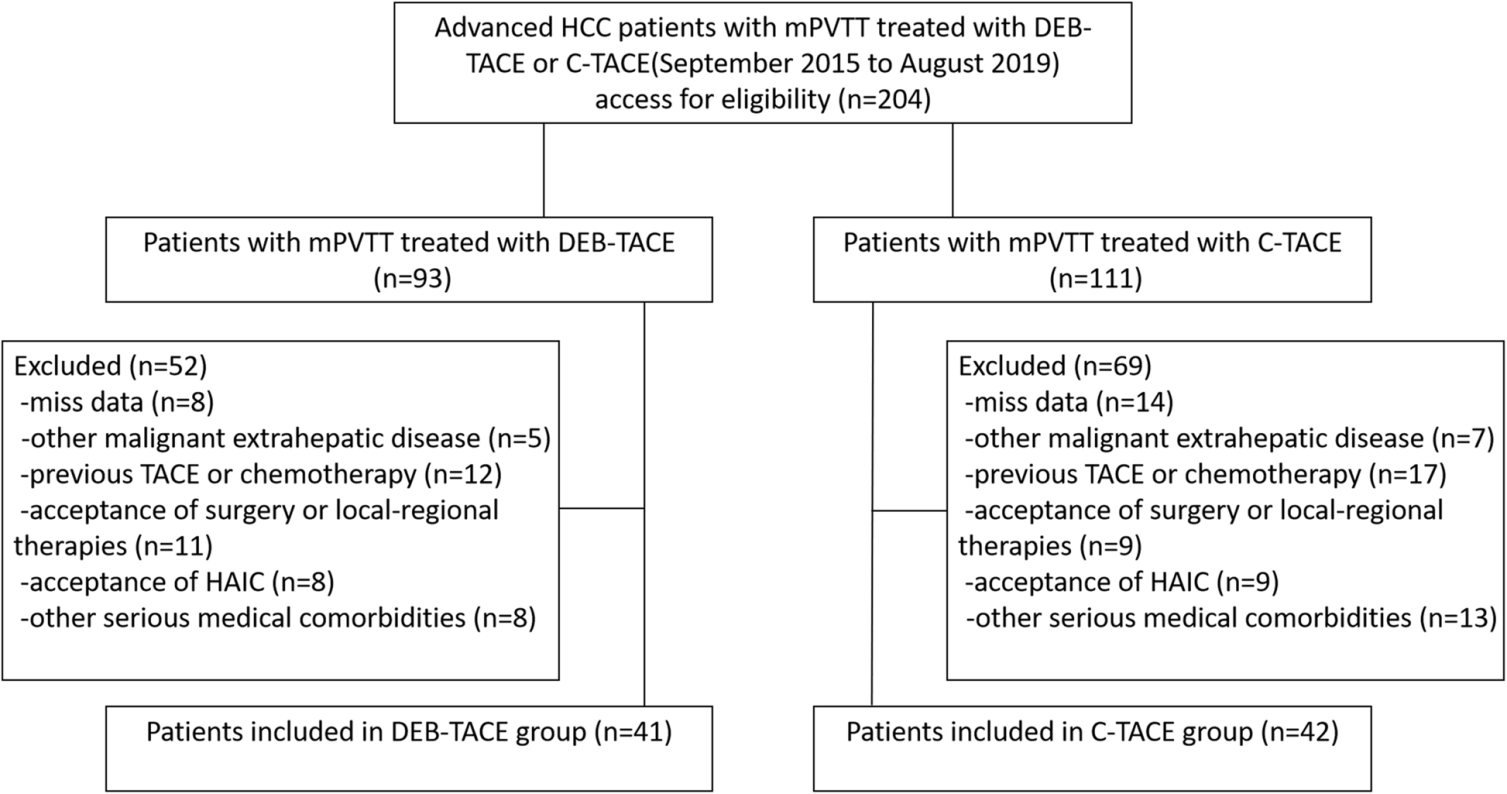
Flow diagram shows exclusion criteria. HCC: hepatocellular carcinoma; mPVTT:main portal vein tumor thrombus; DEB-TACE: Drug Eluting Beads-TACE; C-TACE: Conventional-TACE; HAIC: hepatic arterial infusion chemotherapy

From September 2015 to October 2021, 83 consecutive HCC patients with mPVTT who underwent DEB-TACE or C-TACE were included, and 121 patients were excluded from this research based on the exclusion criteria. Finally, forty-two patients were in the DEB-TACE group, and 41 were in the C-TACE group (Fig. 2). The median follow-up period was 12.0 months (range, 2–35 months) in the DEB-TACE group and 5.0 months (range, 2–28 months) in the C-TACE group. Twenty-nine(69.0%) patients in the DEB-TACE group and 40(97.6%) in the C-TACE group died during the observation period. The baseline characteristics between these two groups were not significantly different (Table 1).

**Table 1:** Baseline patient characteristics

	**DEB-TACE (*n* = 42)**	C- **TACE (*n* = 41)**	***P*-Value**
**Sex (Male/Female)**	40/2	37/4	0.649*
**Age**	52.7 ± 12.1	53.0 ± 10.3	0.908
**Child–Pugh Score**			0.447^#^
**A**	32	34	
**B**	10	7	
**Liver Functions**			
**TBILI (umol/L)**	19.1 ± 13.1	20.1 ± 12.7	0.721
**Albumin (g/L)**	37.6 ± 4.8	37.2 ± 6.8	0.781
**PT-sec (sec)**	14.3 ± 1.0	14.5 ± 1.1	0.483
**AFP Value**			0.447^**#**^
**≤ 400 ng/ml**	17	20	
**> 400 ng/ml**	25	21	
**Capsular Infiltration**			0.446^#^
**Non-infiltration**	15	18	
**Infiltration**	27	23	
**Tumor maximum size**			0.555^#^
**≤ 5 cm**	8	10	
**> 5 cm**	34	31	
**Tumor Numbers**			0.383^#^
**Single**	7	10	
**Multiple**	35	31	
**APS**			0.591^#^
**Present**	16	18	
**Absent**	26	23	
**HVI**			0.395^#^
**Present**	26	29	
**Absent**	16	12	
**EHS**			0.584^#^
**Present**	12	14	
**Absent**	30	27	

*AFP* alpha fetoprotein, *APS* ArterioPortal Shunt, *HVI* Hepatic Vein Invasion, *EHS* Extra Hepatic Spread

^*^continuity corrections was used

^#^Pearson Chi-square was used

Twenty-eight (66.7%) patients in DEB-TACE group and 23 (56.1%) patients in C-TACE group underwent repeated TACE procedures, with a mean of 2.3 ± 1.3 times (range, 1–6) in the DEB-TACE group and 2.1 ± 1.3 times (range, 1–7) in the C-TACE group (*P* = 0.421). On average, there were 1.6 ± 0.88 (range, 1–4) DEB-TACE procedures in DEB-TACE group and 0.1 ± 0.3 (range, 0–1) procedure DEB-TACE procedures in C-TACE group (*P* < 0.001), which represented 5 patients only. Sixteen patients (38.1%) accepted TKI in DEB-TACE group, and 14 patients (34.1%) were in C-TACE group (*P* = 0.708).

### Liver function change and AEs after the first TACE procedure

Liver function changes within 7 days after the TACE procedure in both groups are shown in Table 2. The liver function in the patients with liver function changes returned to baseline in 1 month follow-up. AEs related to the first TACE procedure were recorded in Table 3, and there was no significant difference between these 2 groups. Furthermore, there were 11 patients (fever: 2, abdominal pain: 4, GI bleeding: 3 and liver abscess: 2) with grade 3 or above AEs in DEB-TACE group, meanwhile 18 patients (fever: 3, abdominal: 6, GI bleeding: 6 and liver abscess: 3) with grade 3 or above AEs in C-TACE group (*P* = 0.091). No procedure-related death were found after first TACE procedure within 1 month in both groups.

**Table 2:** Liver function changes 7 days after first TACE procedure for the two groups

	**DEB-TACE group**			**C-TACE group**		
	**Baseline**	**After**	***P*-Value**	**Baseline**	**After**	***P*-Value**
**AST**	85.7 ± 53.1	156.1 ± 142.3	< 0.001	78.6 ± 45.1	409.3 ± 516.5	< 0.001
**ALT**	56.3 ± 31.4	134.6 ± 112.7	0.004	58.7 ± 26.0	341.5 ± 443.9	< 0.001
**TBILI**	19.1 ± 13.1	25.8 ± 21.6	0.090	20.1 ± 12.7	38.4 ± 28.7	< 0.001
**ALB**	37.6 ± 4.8	34.9 ± 3.9	0.006	37.2 ± 6.8	32.8 ± 4.1	0.001
**PT-sec**	14.3 ± 1.0	14.4 ± 1.1	0.718	14.5 ± 1.1	15.4 ± 1.3	0.001

*AST* Aspartate aminotransferase, *ALT* Alanine aminotransferase, *TBILI* Total bilirubin, *ALB* Albumin, *PT* Prothrombin time

**Table 3:** AEs assessment after 1^st^ TACE procedure of two groups

Adverse Events	DEB-TACE group (*n* = 42)	TACE group (*n* = 41)	*P*-Value
**Fever**	27 (64.2)	31 (75.6)	0.261^**#**^
**Abdominal pain**	21 (50.0)	29 (70.7)	0.054^**#**^
**Vomiting**	4 (9.5)	8 (19.5)	0.196^**#**^
**New ascites**	5 (11.9)	7 (17.1)	0.503^**#**^
**GI bleeding**	3 (7.1)	6 (14.6)	0.457^**$**^
**Ischemic cholecystitis**	2 (4.7)	6 (14.6)	0.249^**$**^
**Liver abscess**	2 (4.7)	3 (7.3)	0.978^**$**^
**Hepatic arterial dissection**	0 (0)	1 (2.4)	0.494*
**Pulmonary/cerebral embolization**	0 (0)	0 (0)	…

Data are numbers of events. Data in parentheses are percentages

^#^Pearson Chi-square was used. ^$^ Continuity correction was used

^*^Fisher exact test was used

GI-gastrointestinal

### Tumor response and time to tumor progression

**Fig. 3:**
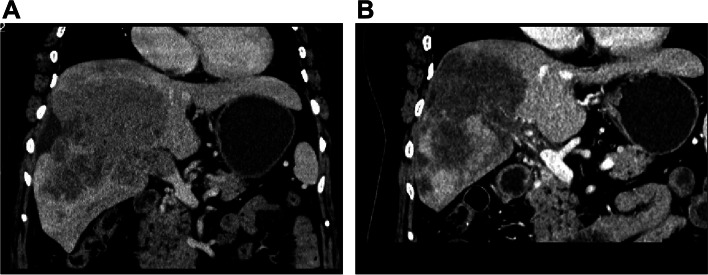
Tumor response of A 66-year-old female HCC patients with mPVTT in the DEB-TACE group. **A** a coronal CT scan showed the tumor thrombus in the main portal vein. **B** coronal CT scan showed the PR. HCC = hepatocellular carcinoma; mPVTT = main portal vein tumor thrombus; PR = partial response

**Fig. 4:**
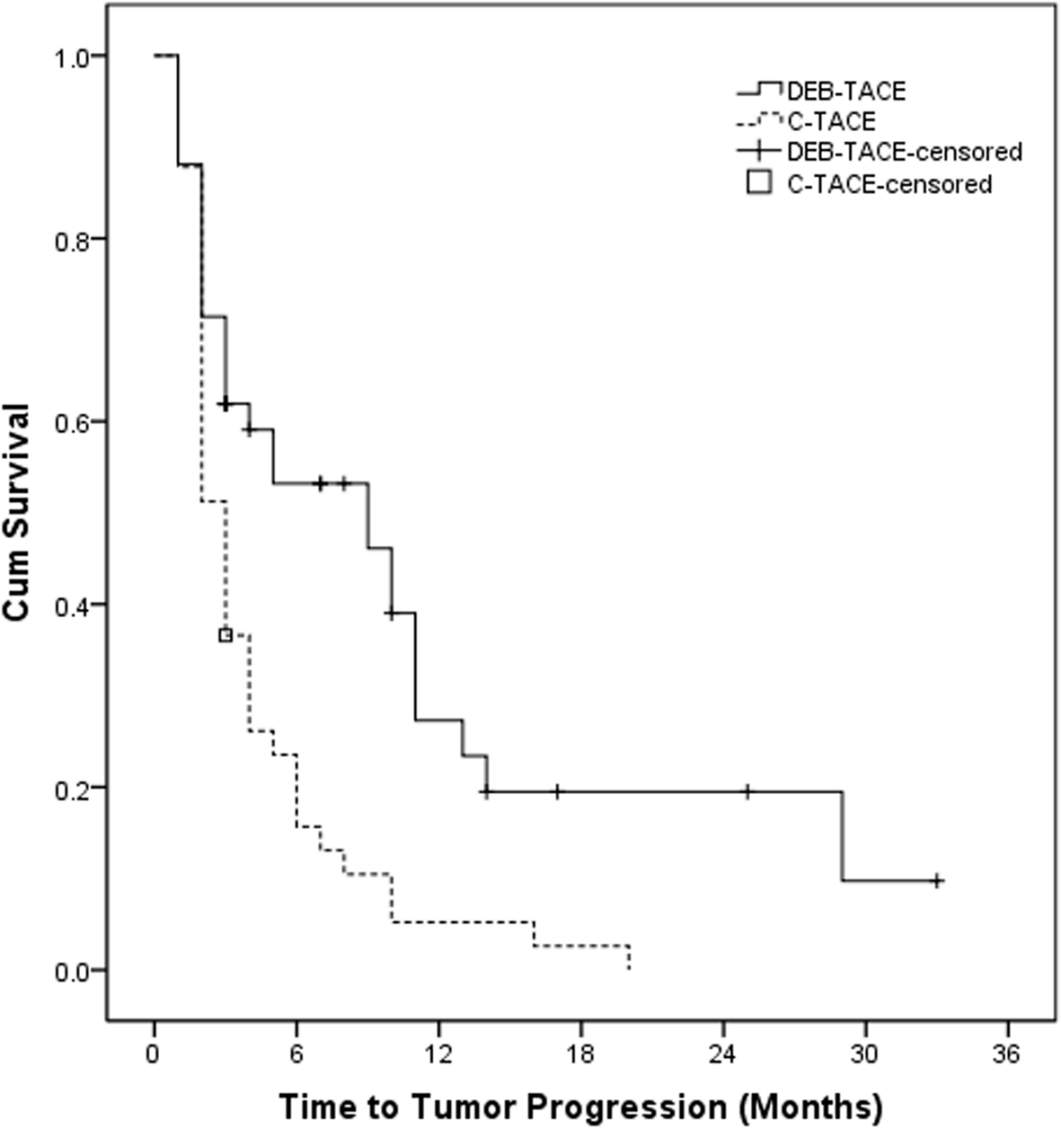
Kaplan–Meier curves of TTP in HCC patients with mPVTT who underwent DEB-TACE or C-TACE

The tumor response after the initial TACE procedure in both groups was recorded (Figs. 1E, F, 3B and Supplement Figs. 1C, D, 2A, B, C). DCR in the DEB-TACE group (CR = 3, PR = 19, SD = 10, PD = 10) were significantly better than those in the C-TACE group (CR = 0, PR = 9, SD = 13, PD = 19) (76.2% vs. 53.7%, *P* = 0.031). The median TTP was 9.0 months (95% CI: 3.8–14.2 months) in DEB-TACE group and 3.0 months (95% CI: 2.5–3.5 months) in C-TACE group (*P* < 0.001) (Fig. 4).

### Overall survival and subgroup analysis

**Fig. 5:**
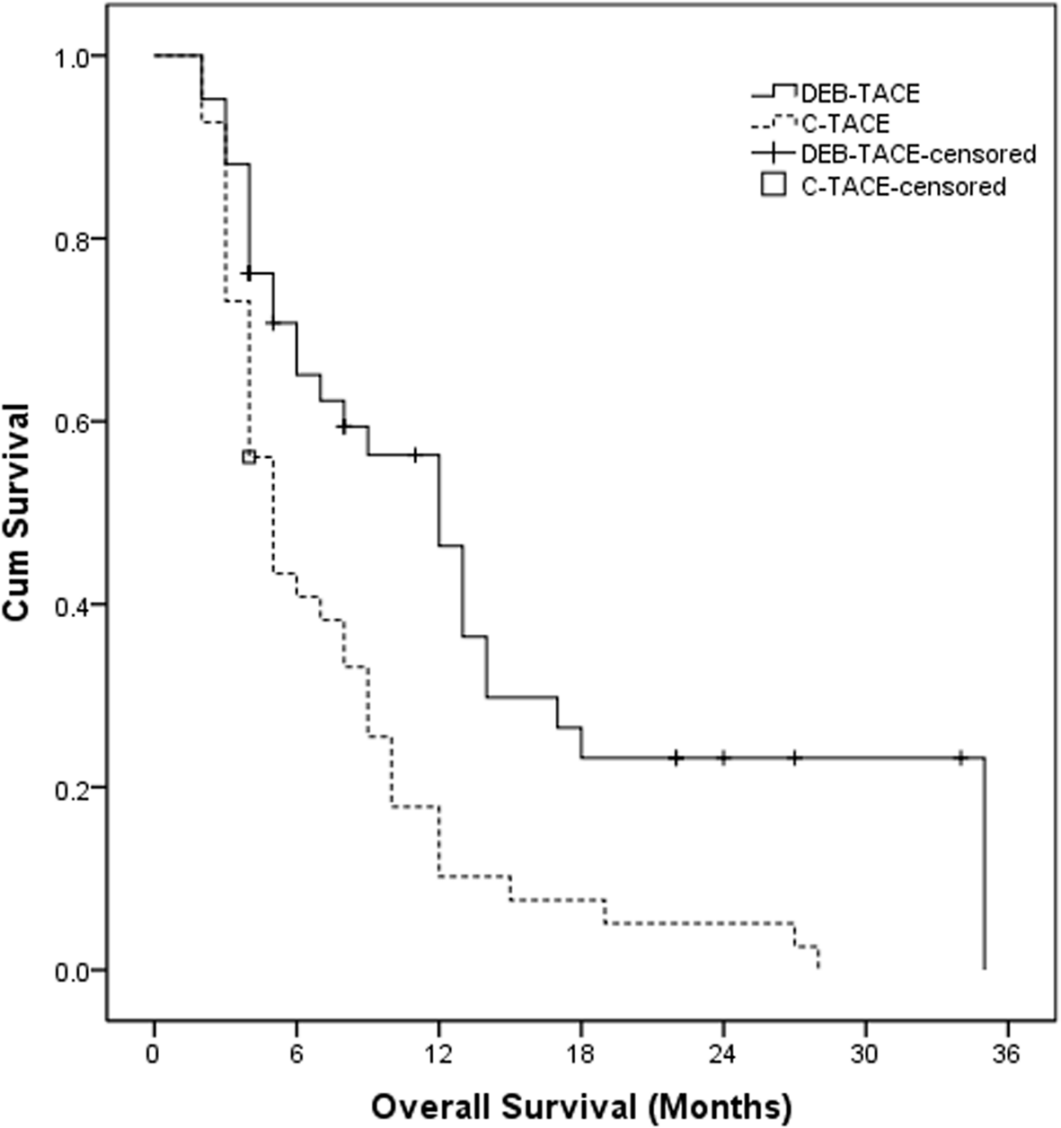
Kaplan–Meier curves of OS in HCC patients with mPVTT who underwent DEB-TACE or C-TACE

The median OS was 12.0 months (95% CI: 8.6–15.4 months) in DEB-TACE group and 5.0 months (95% CI: 3.8–6.2 months) in C-TACE group (*P* < 0.001) (Fig. 5). The OS between DEB-TACE group and C-TACE group was significantly different.when it stratified according to the Child–Pugh class, tumor characteristics (AFP value, APS, HVI, EHS, maximum diameter, capsular infiltration and tumors number) and tumor response after initial TACE procedure. The median OS in patients with Child–Pugh class B (*P* = 0.997), maximum diameter ≤ 5 cm (*P* = 0.254), single tumor lesion (*P* = 0.668) and tumor response with PD (*P* = 0.590) had no significant difference between these two groups in subgroup analysis (Table 4).

**Table 4:** Subgroup analysis of OS in mPVTT patients treated with DEB-TACE or C-TACE

Factors	Median OS in DEB-TACE (Months)	Median OS in C-TACE (Months)	*P*-Value
Child–Pugh			
A	13.0 (11.5–14.5)	4.0 (3.0–5.0)	< 0.001
B	9.0 (2.8–15.2)	10.0 (4.4–15.6)	0.997
AFP value			
≤ 400 ng/ml	14.0 (8.9–19.1)	8.0 (3.9–12.1)	0.033
> 400 ng/ml	12.0 (5.2–18.8)	4.0 (2.5–5.5)	0.001
APS			
Present	5.0 (3.0–7.0)	3.0 (2.1–3.9)	0.040
Absent	14.0 (11.4–16.6)	9.0 (5.7–12.3)	0.001
HVI			
Present	12.0 (5.7–18.3)	4.0 (3.0–5.0)	0.003
Absent	13.0 (9.1–16.9)	9.0 (4.8–13.2)	0.058
EHS			
Present	12.0 (4.1–19.6)	4.0 (3.1–4.9)	0.016
Absent	12.0 (8.0–16.0)	6.0 (3.1–8.9)	0.007
Maximum Diameter			
≤ 5 cm	14.0 (12.0–16.0)	12.0 (2.6–21.4)	0.254
> 5 cm	12.0 (6.4–17.6)	5.0 (3.9–6.1)	< 0.001
Capsular Infiltration			
Non-infiltration	18.0 (8.2–27.8)	9.0 (3.8–14.2)	0.001
Infiltration	8.0 (4.2–11.8)	4.0 (2.4–5.6)	0.012
Tumor Numbers			
Single	14.0 (1.0–27.0)	4.0 (2.5–5.5)	0.668
Multiple	12.0 (7.5–16.5)	5.0 (2.8–7.2)	< 0.001
Tumor Response			
CR + PR + SD	14.0 (12.2–15.8)	9.0 (6.3–11.7)	0.001
PD	4.0 (3.1–4.9)	4.0 (3.4–4.6)	0.590

*AFP* alpha fetoprotein, *APS* ArterioPortal Shunt, *HVI* Hepatic Vein Invasion, *EHS* Extra Hepatic Spread, *CR* Complete Response, *PR* Partial Response, *SD* Stable Disease, *PD* Progression Disease

### Uni- and Multivariate Analysis

Univariable analyses and multivariate analysis was performed including different variables. And DEB-TACE (*P* = 0.001), APS absence (*P* = 0.019), capsular non-infiltration (*P* = 0.015) and tumor response with CR + PR + SD (*P* < 0.001) were identified as independent prognostic factors for OS by multivariate analysis (Table 5).

**Table 5:** Univariate and multiple analysis of prognostic factor for OS

	**Univariate Analysis**			**Multivariate Analysis**	
Variable	**No. of Patients (*n* = 83)**	**Median OS**	***P*-Value**	Hazard Ratio *	***P*-Value**
**Methods**			< 0.001		0.001
** DEB-TACE**	42	12.0 (8.7–15.3)		1	
** C-TACE**	41	5.0 (3.8–6.2)		2.4 (1.4–4.1)	
**Child–Pugh**			0.663		
** A**	66	7.0 (3.8–10.1)			
** B**	17	9.0 (1.7–16.3)			
**AFP value**			0.297		
** ≤ 400**	37	9.0 (5.5–12.5)			
** > 400**	46	6.0 (4.2–7.8)			
**APS**			< 0.001		0.019
** Present**	34	4.0 (2.7–5.3)		1.9 (1.1–3.2)	
** Absent**	49	12.0 (9.6–14.4)		1	
**HVI**			0.133		
** Present**	55	5.0 (3.0–7.0)			
** Absent**	28	10.0 (5.8–14.2)			
**EHS**			0.831		
** Present**	26	5.0 (0.3–9.7)			
** Absent**	57	8.0 (5.1–10.9)			
**Maximum Diameter**			0.086		0.456
** ≤ 5 cm**	18	12.0 (5.0–19.0)			
** > 5 cm**	65	6.0 (3.8–8.2)			
**Capsular Infiltration**			0.001		0.015
** Non-infiltration**	33	12.0 (9.1–14.9)		1	
** Infiltration**	50	5.0 (3.5–6.5)		1.9 (1.1–3.3)	
**Tumor Numbers**			0.533		
** Single**	17	13.0 (0.0–28.2)			
** Multiple**	66	8.0 (5.6–10.4)			
**DCR**			< 0.001		< 0.001
** CR + PR + SD**	54	12.0 (9.3–14.7)		1	
** PD**	29	4.0 (3.4–4.6)		4.0 (2.2–7.3)	
**Follow-up TKI**			0.155		
** Yes**	30	9.0 (5.4–12.6)			
** No**	53	5.0 (3.0–7.0)			

*AFP* Alpha fetoprotein, *APS* ArterioPortal Shunt, *HVI* Hepatic Vein Invasion, *EHS* Extra Hepatic Spread, *TKI* Tyrosine Kinase Inhibitor

## Discussion

The prognosis of patients with mPVTT is extremely poor, and obstruction of the portal vein can result in deteriorated liver function, cause refractory ascites, and induce variceal bleeding. The reported median OS for untreated HCC patients with mPVTT was only 2.7 months [16]. Sorafenib has been the recommended treatment option for HCC with mPVTT based on several guidelines, even though the OS is only 3–6 months [17, 18]. Other systemic therapy (atezolizumab and bevacizumab, which was proven better prognosis than sorafenib in HCC patients) is need further investigation in mPVTT patients [19].

C-TACE has been reported to achieve clinical benefit (median OS: 5.6 months) in HCC patients with mPVTT, and previous research had also reported that C-TACE could improve the OS in patients with PVTT compared to system therapy [8, 9]. However, the risk of deteriorating liver function due to ischemic liver damage limited the usage of C-TACE [20, 21]. DEB-TACE is a relatively new technology commonly used in BCLC-B HCC, and is considered less harmful to liver function and lower rate of doxorubicin-related side effects [22, 23]. The present study also proved that serum AST, ALT, ALB, TBILI and PT within 7 days after initial C-TACE procedure were significantly different from baseline values. On the other hand, only serum AST, ALT and ALB in the DEB-TACE group showed significant difference. Consequently, DEB-TACE might be more safely performed in mPVTT patients, and it is recommended to have a prospective randomized control trial (RCT) to further investigate the safety benefit.

DCR of C-TACE in HCC patients with mPVTT was reported poor (46.2%-50%) [24, 25]. There might be several reasons: 1) injected lipiodol/drug emulsion is prepared extemporaneously and might be unstable, poor lipiodol retention in tumor and washout would also lower the antitumor effect of C-TACE procedure [26–28]; 2) IRs’ technique and experience would affect lipiodol droplet size and viscosity for optimal embolization of C-TACE [29, 30], which might affect the C-TACE reproducibility and efficiency. While DEB-TACE has been proved to gain a better DCR (76.2%) and prolong TTP in mPVTT patients in this research. Our group inferred that the features of DEB-microspheres and superselective embolization during the DEB-TACE treatment allow for optimal embolization of the feeding artery of HCC lesions and mPVTT, which might lead to the occluding tumor vessels more effectively [31–33]. And Imai Y’s study proved that DEB-microspheres could be found in the PVTT without recanalization [34]. Similar results were proven in the subgroup analyses (more advanced disease, including Child–Pugh B, ECOG 1, bilobar or recurrent disease) of the PRECISION-V trial, which showed that the DCR and OS were better in DEB-TACE [23].

Our research showed that DEB-TACE might significantly improve the OS of HCC patients with mPVTT with similar TACE number of TACE procedure as compared to C-TACE group. These founding was also proven in several researches [35, 36]. The smaller size DEB-microspheres (30–60 um or 50–100 um) was supposed to be main cause of the good therapeutic effect in these patients, which was proven to be linked with higher survival rate and lower complications compared to bigger size DEB-microspheres in previous research [37]. Meanwhile, the slow release of anticancer drugs from DEB-TACE enables a sustained antitumor effect, which might also be contributed to the longer TTP and OS in DEB-TACE group [38]. Moreover, the superselective embolization of the mPVTT was always done as first steps during the TACE procedure was the important reason for the good clinical result, which might lead to the necrosis of the mPVTT and good tumor response in this research.

Interestingly, we found that mPVTT patients with Child–Pugh class B (*P* = 0.997), absence of HVI (*P* = 0.058), maximum diameter ≤ 5 cm (*P* = 0.254), single tumor lesion (*P* = 0.668) and tumor response with PD (*P* = 0.590) did not gain survival benefit by initial DEB-TACE compared to C-TACE procedure. Relatively low number of these patients in the subgroup analysis (Child–Pugh class B: 17; single tumor lesion: 17; maximum diameter ≤ 5 cm: 17) might led to these results in survival analysis. Furthermore, it has been suggested that treating HCC is to preserve liver function as much as possible with effective intrahepatic tumor control. And these two factors are the most important prognostic factors in patients with HCC, which was well demonstrated, even with extrahepatic metastasis [39]. In conclusion, we suggested that patients with above situation, especially patients with Child-Pugu Class B, should not be considered candidates for DEB-TACE, which is much more expensive than C-TACE.

Additionally, we found that the absence of APS and capsular non-infiltration were independent prognostic factors for longer OS. It has been reported that APS might affect the safety of C-TACE because lipiodol/drug emulsions can be easily washed out through shunts instead of retaining within tumors, which might result in the shorter survival of advanced HCC patients [40]. DEB-microspheres was reported to allow for sustained drug delivery and simultaneous permanent embolization, might improve the treatment effect, even in patients with APS present [23, 41]. Meanwhile, the capsular non-infiltration is another predictive factor. Several studies also reported that prognosis of patients with capsular non-infiltration is poor, and the therapeutic modality is limited [42, 43].

There were several limitations in this research: 1) retrospective, and initial therapeutic options (DEB-TACE vs. C-TACE) in patients with mPVTT were individually determined on the basis of the attending physician’s experience and preference, which likely led to selection bias in our population. However, the bias was justified by similar baseline characteristics between these two groups. During patients’ follow-up visits, IRs encouraged the same treatment option for patients but they could still choose DEB-TACE or C-TACE by themselves. Based on our cohort data, only 5 patients from C-TACE group chose DEB-TACE during the follow-up due to unfavorable initial treatment outcome and cost concern of the treatment. The mean number of DEB-TACE was 1.6 ± 0.88 in DEB-TACE group; and that was 0.1 ± 0.3 in the C-TACE group. We believed that the cross-over issue is minor and would not affect our study results. 2) The number was limited (42 in DEB-TACE group and 41 in C-TACE group). The relatively small number of patients most likely led to the difference in the median OS, especially in the subgroup analysis. 3) The BCLC classification recommends TKI as a standard therapy in HCC patients with PVTT. In this study, only 30 patients (36.1%) accept additional TKI post-transarterial treatment and the additional TKI was not identified as a prognostic factor of OS. Consequently, an adequately powered prospective, randomized trial of DEB-TACE in mPVTT patients is necessary to confirm our findings, especially compared with TKI alone. Meanwhile, transarterial radioembolization (TARE) was reported to improve the OS in advanced HCC patients with PVTT compared to TKI [44]. Because it was supposed to produce much lesser embolic effects than TACE, PVTT is not a contraindication for TARE. However, at the time of our study, TARE was not registered in China and there was no such treatment option for patients with mPVTT. Therefore, a RCT of DEB-TACE compared with TARE in patients with mPVTT is needed to be further studied.

## Conclusion

DEB-TACE might yield a promising outcome in HCC patients with mPVTT. The longer OS of mPVTT patients was associated with DEB-TACE procedure, absence of APS, non-infiltration and better DCR.

## Supplementary Information

**Additional file 1.**

## Data Availability

The datasets used or analysed during the current study are available from the corresponding author on reasonable request.

## Abbreviations

HCC: Hepatocellular carcinoma
mPVTT: Mail portal vein tumor thrombus
DEB-TACE: Drug-eluting bead-transarterial chemoembolization
C-TACE: Conventional-transarterial chemoembolization
AEs: Adverse events
DCR: Disease control rate
CR: Complete response
PR: Partial response
SD: Stable disease
PD: Progression disease
APS: Arterioportal Shunt
HVI: Hepatic vein invasion
EHS: Extra hepatic spread,
TTP: Time to tumor progression
OS: Overall survival
