# Supplementary material for: Safety, efficacy, and survival of drug-eluting beads-transarterial chemoembolization vs. conventional-transarterial chemoembolization in advanced HCC patients with main portal vein tumor thrombus
Source: Cancer Imaging. 2023 Jul 22;23:70. doi: 10.1186/s40644-023-00581-8 (PMC10362718; doi:10.1186/s40644-023-00581-8)
Supplement: Supplementary file 1 — Additional file 1. [file 40644_2023_581_MOESM1_ESM.doc]

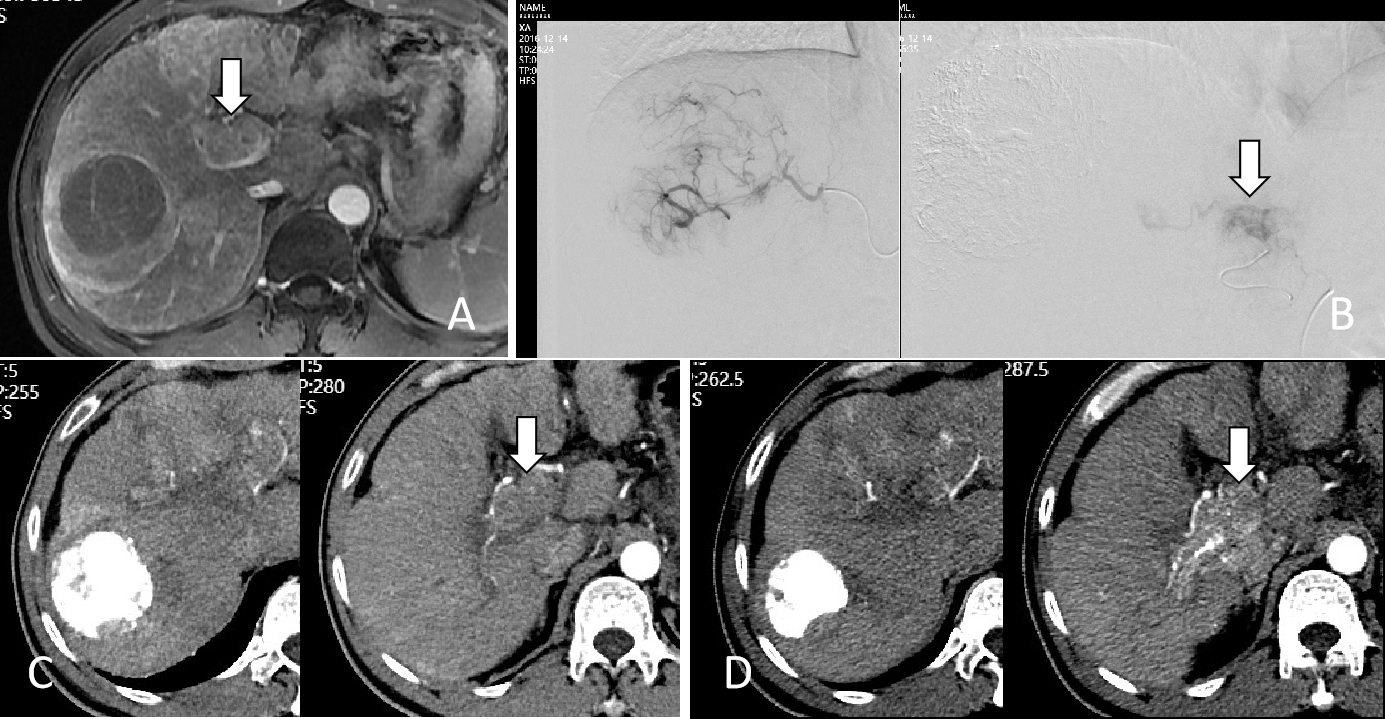


**Supplement Figure 1:** superselective embolization of C-TACE procedure, and 1 month and 3 months follow-up contrast-enhanced CT scan.

Note: A 52-year-old male HCC patient: (A) contrast-enhanced MRI showed HCC lesion in right lobe with mPVTT (white arrow). (B) superselective embolization of the HCC lesion and mPVTT (white arrow) during the C-TACE procedure. (C,D) contrast-enhanced CT scan follow-up showed PR at 1 month and PD at 3 months (white arrow: mPVTT).


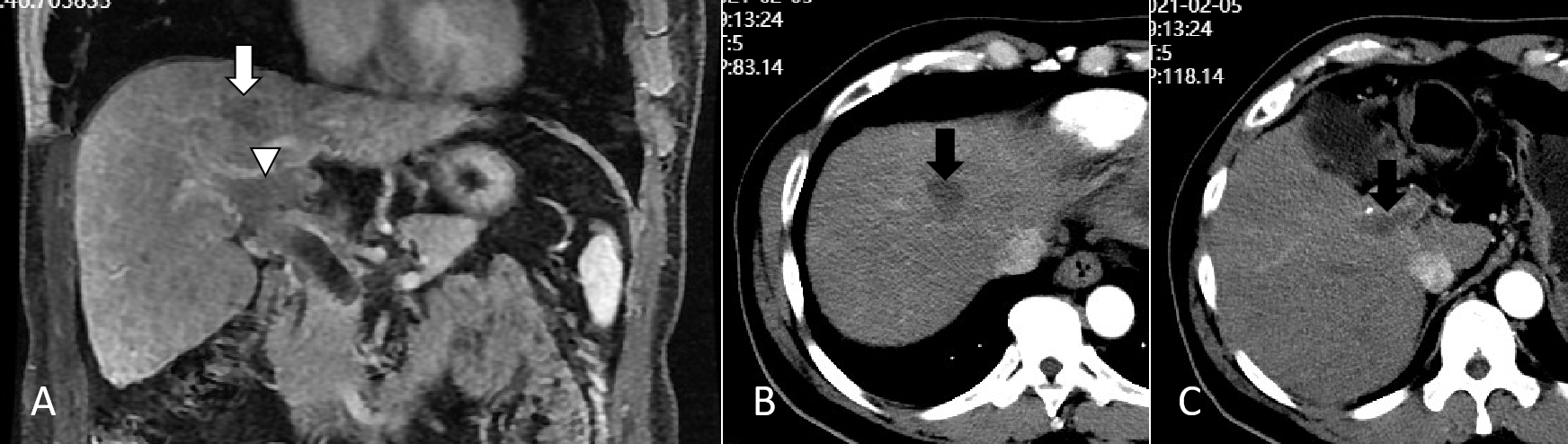


Supplement Figure 2: a 53-year-old male HCC patient: (A) contrast-enhanced MRI showed HCC lesion in right lobe (white arrow) with mPVTT (white arrow head). (B, C) contrast-enhanced CT scan follow-up showed CR in 5 week (white arrow: HCC lesion and mPVTT)
